# “We’re categorized in these sizes—that’s all we are”: uncovering the social organization of young women’s weight work through media and fashion

**DOI:** 10.1186/s12889-022-13607-w

**Published:** 2022-06-15

**Authors:** Alexa R. Ferdinands, Tara-Leigh F. McHugh, Kate Storey, Kim D. Raine

**Affiliations:** 1 School of Public Health, Centre for Healthy Communities, University of Alberta, 3-300 Edmonton Clinic Health Academy, 11405 – 87 Ave., Edmonton, AB T6G 1C9 Canada; 2 Faculty of Kinesiology, Sport, and Recreation, University of Alberta, Edmonton, Canada

**Keywords:** Institutional ethnography, Weight stigma, Fashion, Media, Women, Public health

## Abstract

**Background:**

For decades, dominant weight discourses have led to physical, mental, and social health consequences for young women in larger bodies. While ample literature has documented *why* these discourses are problematic, knowledge is lacking regarding *how* they are socially organized within institutions, like fashion and media, that young women encounter across their lifespan. Such knowledge is critical for those in public health trying to shift societal thinking about body weight. Therefore, we aimed to investigate how young women’s weight work is socially organized by discourses enacted in fashion and media, interpreting work generously as any activity requiring thought or intention.

**Methods:**

Using institutional ethnography, we learned from 14 informants, young women aged 15–21, in Edmonton, Canada about the everyday work of growing up in larger bodies. We conducted 14 individual interviews and five repeated group interviews with a subset (*n* = 5) of our informants. A collaborative investigation of weight-related YouTube videos (*n* = 45) elicited further conversations with two informant-researchers about the work of navigating media. Data were integrated and analyzed holistically.

**Results:**

Noticing the perpetual lack of larger women’s bodies in fashion and media, informants learned from an early age that thinness was required for being seen and heard. Informants responded by performing three types of work: hiding their weight, trying to lose weight, and resisting dominant weight discourses. Resistance work was aided by social media, which offered informants a sense of community and opportunities to learn about alternative ways of knowing weight. However, social media alleging body acceptance or positivity content often still focused on weight loss. While informants recognized the potential harm of engagement with commercial weight loss industries like diet and exercise, they felt compelled to do whatever it might take to achieve a “normal woman body”.

**Conclusions:**

Despite some positive discursive change regarding body weight acceptance in fashion and media, this progress has had little impact on the weight work socially expected of young women. Findings highlight the need to broaden public health thinking around how weight discourses are (re)produced, calling for intersectoral collaboration to mobilize weight stigma evidence beyond predominantly academic circles into our everyday practices.

**Supplementary Information:**

The online version contains supplementary material available at 10.1186/s12889-022-13607-w.

## Introduction

“The appearance of the body as object is a practice learned in childhood ([1] p. 145)”. 

Western society’s critical gaze on women’s bodies is rarely questioned. In Canadian sociologist Dorothy Smith’s seminal work, *Texts, Facts, and Femininity*, she introduced readers to a novel way of understanding women’s bodies: as a never-ending, text-mediated project [1]. One piece of this appearance-focused project pertains to weight. Smith explained, “being fat breaks with the paradigmatic image of the ‘reed thin’ woman of the texts of femininity … the fat woman is not ‘read’ as feminine” ([1] p. 136). Smith drew attention to how images of women’s bodies—on screens, magazine covers, or breathing bodies on the street—are objectified and read as texts, their messages replicated across time and space. These texts instruct women from a young age why and how to manicure their bodies, telling them what body weights are not just ideal, but also “normal”. Further, these texts, reflecting dominant discourses concerning femininity, health, and weight, commonly link virtue and morality with body weight [2]—a public health issue perpetuated by scientific literature [3]. Notably, the blaming and shaming of people in larger bodies (i.e., weight stigma) is gendered in Western countries like Canada [4–6]. Generally, girls’ and women’s bodies are objectified and stigmatized to a greater extent than boys’ and men’s bodies. However, boys and men are increasingly facing similar pressures, although to achieve a lean, muscular, mesomorphic shape rather than the socially desirable ectomorphic shape for girls and women [7–9].

Ample research has demonstrated the stigmatization of people of all ages based on their weight, fuelled by prevailing societal discourses that weight is personally controllable, that weight and health are directly correlated, and that weight is reflective of moral character [10–12]. Higher weight young people face multiple forms of weight stigma in all domains of life, such as social exclusion among peers, bullying from family members, and dismissal of health concerns from medical professionals [13–16]. Young people are also surrounded by mass media messaging reinforcing fat stereotypes [17–19]. From a public health perspective, the cumulative health impact of weight-related microaggressions and discrimination across the lifespan is concerning [20]. For more than five decades [10, 21], literature has documented the serious physical, mental, and social health consequences of weight stigma, such as depression, anxiety, disordered eating, negative body image, and weight cycling [22–24].

Encouragingly, more and more public health scholars are questioning the assumption that fatness inherently causes health issues and is a problem to be “fixed,” acknowledging that attempting to do so may inflict more harm to people’s health than good. Smith’s generous conception of work is a useful tool for deconstructing this assumption [25, 26]. Originally conceptualized by the Marxist-feminist group, Wages for Housework, the term “work” includes any paid or unpaid task that requires time, effort, and intent [25, 26]. Such mundane physical, mental, and emotional tasks may not be recognized as “work” by the person doing them (indeed, they may not even be conscious of their knowledge required for doing this work) [25, 26]. However, identifying these tasks and showing how they are coordinated to happen can help public health professionals detect and act on levers for change.

In trying to conform to societal ideals, people in fat bodies do different work than people in thin bodies. One such type of work relates to clothes shopping and getting dressed. A growing body of literature has drawn attention to the limited clothing options for heavier adult women, although literature is lacking on whether clothing options for larger girls are equally limited [27–31]. Girls’ and women’s weight work when shopping is socially organized by texts, such as Smith’s aforementioned “reed thin” woman ([1] p. 136). These texts indoctrinate a fear of fatness in women from birth, as girls and women are taught to pursue thinness no matter the cost, monetary or otherwise. In observing the lack of fashionable clothing for bigger bodies, they recognize they must lose weight to fit in.

Another form of girls’ and women’s work pertains to navigating weight loss marketing, and fat-shaming content more generally, in mass media and social media (hereafter referred to collectively as “media”) [32–36]—an especial concern for young people who are vulnerable to its messaging. Youths’ cognitive development is limited; they are prone to perceiving such marketing and misinformation as objective and truthful [37, 38]. In addition to images, videos, and text endorsing the thin ideal, the anonymity tied to platforms like Instagram, Facebook, Twitter, and YouTube, and the rarity with which policies prohibiting hate speech on these platforms are enforced, have fueled hateful dialogue towards people in larger bodies [35].

Despite substantial evidence on the harms of weight stigma, minimal progress has been made in reducing it at a societal level [39]. Most weight stigma intervention research has concentrated on changing individuals’ attitudes, beliefs, and behaviors [40–42]. Fewer efforts have addressed how weight stigma manifests from broader social conditions, like institutional policies and practices [43, 44]—essential knowledge for effecting lasting public health change. One way to deepen our understanding of how weight stigma happens is to examine the discourses embedded in the institutions that people encounter in their everyday lives. These institutions, interpreted as complexes of social relations as opposed to brick-and-mortar structures [26], include fashion and media. Despite their clear potential for overlap, no research to our knowledge has examined these institutions in tandem to examine how they mutually reinforce one another to create stigmatizing conditions for young women to grow up in. We focused on women’s experiences because women are disproportionately subject to greater weight-related scrutiny than men in Canada [5, 45, 46], where this research took place. Overall, the purpose of this exploratory study was to provide an overview of the social organization of young women’s weight work that is coordinated by text-mediated discourses in fashion and media.

## Methods

### Institutional ethnography

We used Smith’s institutional ethnography (IE) as our method of inquiry [26]. With its origins in second-wave feminism in North America [47], IE is well-suited to explicating gendered public health issues. Institutional ethnographers explore how people’s everyday activities are coordinated and regulated by institutional policies and practices [26]. They assume what happens locally to be coordinated by ruling relations, which Campbell and Gregor describe as the “socially-organized exercise of power that shapes people’s actions and their lives,” frequently without our knowledge ([48] p. 32). This power often manifests in the form of text-mediated discourses connecting people across time and space. The term “social organization” is a cornerstone of IE, referring to the “material and reflexive coordination of people’s actions, as observable and reproduced across time and place” ([49] p. 618).

Epistemologically, IE is grounded in anti-positivism, valuing reflexive and experiential knowledge over objectivity and abstract ideology [49, 50]. Ontologically, institutional ethnographers assume that the social world is created through people’s ongoing practices, which opens the possibility for people to effect change through their actions [49]. IE’s social ontology commits researchers to shift “agency away from concepts … back into the embodied knower” ([51] p. 5).

An IE investigation begins from an embodied standpoint; that is, the social position of those whose interests we aim to serve [26]. Here, standpoint informants were young women who had grown up in bigger bodies. To conduct research from their standpoint, the analysis had to be anchored in the material conditions of their lives (i.e., their work). Work is a fundamental concept within IE [25]. IE researchers look for “traces of ruling relations within the descriptions of everyday work—those occasions when the work done at the standpoint does not seem to be supporting the interests of the people *there”* ([52] p. 2, emphasis in original)*.* IE is pragmatic; rather than abstracting into theory, we empirically trace how something is happening. Data employed in this manuscript stem from a broader IE project on the social organization of young people’s work of growing up in larger bodies [53, 54]. We describe the three data sources (individual interviews, group interviews, YouTube investigation) used below.

### Individual interviews

In IE, “each interview provides an opportunity to learn about a particular piece of the extended relational chain, to check the developing picture of the coordinative process, and to become aware of additional questions that need attention” ([55] p. 23). After obtaining university ethics approval, we employed various strategies to purposefully recruit standpoint informants in Edmonton, Canada, including drawing upon the social networks of Obesity Canada [56] (a charitable organization aiming to reduce weight discrimination); posting advertisements at health clinics, cafes, libraries, recreation centres; and advertising through university email listservs. Recruitment ceased when we achieved saturation; that is, when new ideas or topics no longer emerged in our conversations. We recruited a total of 14 young women with diverse life experiences between the ages of 15–21 who self-identified as having grown up bodies labelled as “overweight” or “obese”. Numerical weights were not obtained as we were interested in their embodied experiences of being fat; precise measurements were unimportant. The lead author (Ferdinands) interviewed each participant once between March and May 2019. The grand tour question posed to informants was “how has it felt to grow up in your body,” to uncover the trajectory of their experiences across the lifespan. This included probing about activities and settings where weight stigma and discrimination might occur, such as when socializing with friends and family, and how informants coped with these experiences. To orient our interviews, she focused on learning about how informants acquired and applied knowledge of their work. For example, when a participant mentioned calorie-tracking, she asked follow-up questions like, “what exactly does calorie-tracking entail? Why do you calorie-track? How do you know how to calorie-track?” Interview questions evolved over time, building upon learnings from previous interviews. Interviews ranged from 43 to 88 min long. Informants were provided with a $30 gift card to thank them for sharing their time and expertise.

### Group interviews

All informants interviewed individually were invited to partake in multiple group interviews for deeper investigation and clarification. Eleven informants cited interest, but due to scheduling difficulties, only five could attend. This subset of five women (Table 1) participated in five group interviews between September 2019 and January 2020, facilitated by Ferdinands. Group interviews occurred on the university campus and were one to two hours long. Conversation topics were emergent, but were intended to deepen and refine questions covered in the individual interviews. Food and beverages were provided at each meeting. A $15 gift card was given to each informant every meeting attended as a token of appreciation.

**Table 1:** Demographic information

Pseudonym	Age (years)	Country/Countries of growing up	Group interview informant?	Informant-researcher?
Amy	21	Canada	No	No
Angela	20	Romania, moved to Canada age 7	Yes	No
Bree	19	Mauritius, moved to Canada age 18	Yes	Yes
Christine	19	Canada	Yes	Yes
Eden	20	Canada	No	No
Elizabeth	19	Canada	No	No
Emma	20	Canada	No	No
Jane	18	Canada	No	No
Jasmine	19	Canada	No	No
Jessica	15	Canada	No	No
Lauren	19	Hong Kong, moved to Canada age 9	Yes	No
Maria	18	Mexico, moved to Canada age 9	No	No
Sarah	18	Kenya, moved to Canada age 17	Yes	No
Sharmeen	15	Canada	No	No

### YouTube investigation

Texts are key to mapping how ruling relations operate across sites [57]. We aimed to expose “how texts enter into, organize, shape, and coordinate people’s doing as they/we participate in the objectifying relations of ruling” ([57] p. 5). YouTube was selected as a textual medium of interest because of how frequently it came up during interviews and because of its audio-visual format which allowed us to assess images and the spoken and written word—valuable for analyzing the discourses embedded in fashion and media.

Our YouTube investigation was not designed as a traditional media analysis. Rather, we aimed to explore YouTube in an emergent, exploratory fashion to elicit conversations with young women about the work associated with navigating media and contextualize the individual and group interview data. In alignment with the collaborative underpinnings of IE, we hired two young women as informant-researchers (who were previous interviewees) to aid in this investigation. They had significantly more expertise navigating YouTube and a keener understanding of how other young women use it than the authors. Together (Ferdinands and informant-researchers), we developed a search strategy and data collection guide (Supplementary File 1). We wanted to examine YouTube in a way that is representative of how young women might *actually* use it, searching for terms identified by informant-researchers as relevant to our research purpose, such as “body positivity,” “weight loss,” and “plus-size,” and using a snowballing technique to follow up on suggested videos in the “up next” section. To ensure we reviewed commonly watched videos, included videos had to have more than one million views each (informally considered a YouTube “milestone” and indicative of a popular video, as per our informant-researchers). Videos were selected by informant-researchers based on the order in which they appeared in the search results (top to bottom). Examples of data collected from videos include demographic information about people in the video, body size language used, and viewer comments. Informant-researchers recorded how the video made them feel about their body and other bodies. Informant-researchers and Ferdinands collected data on five of the same videos to observe how and whether our interpretations differed. In total, informant-researchers collected data on 45 videos between July and August 2019—sufficient for our modest aims of contextualizing interview data and obtaining a broad sense of the video content young women may interact with day-to-day. An hour-long debriefing meeting was held in September 2019 to collectively interpret our results. Informant-researchers and Ferdinands then presented these synthesized findings at a local public health conference.

### Data analysis

In IE, researchers must move beyond conventional ethnographic description to explication; that is, the analytic process of “identifying, tracing, and describing the social relations that extend beyond the boundaries of any one informant’s experiences (or even of all informants’ experiences)” ([48] p. 90). The analytic focal point rested in the social organization of informants’ work. We (all authors and informant-researchers) strove to piece together the puzzle of how weight stigma happens as it does. As is common in qualitative research, data generation and analysis were concurrent, iterative, and reflexive processes [58]. We regularly returned to the data, tying them to preliminary findings to clarify our understanding of work processes [59]. For the individual and group interviews and the YouTube debriefing meeting, Ferdinands audio-recorded and transcribed them verbatim, jotted field notes after each session, and kept a personal journal for documenting and reflecting on the research process. Journal entries also incorporated her reflections on her biases and day-to-day experiences, including debriefings (in person, over video, phone calls, emails, voice memos, and texts) with co-authors (her PhD supervisory committee), other colleagues, and friends. After each informant’s individual interview, Ferdinands wrote a narrative account of their experiences, describing how selected instances of their work were socially organized. Ferdinands listened to audio-recordings and re-read transcripts multiple times, examining the ideas and ideologies embedded in their speech. She then wrote reflexive summaries about what she knew and what she needed to find out.

Indexing was another important systematic process for organizing and working with the data early in the analysis [60]. All data, including reflexive journal notes, were indexed topically using Microsoft Excel, integrated, and analyzed collectively. Indexing, an orienteering tool, was used to stay grounded in the material world and keep informants in view, helping to avoid the tendency to abstract data into theoretical categories [60]. For example, upon establishing the index heading of “shopping for clothes”, Ferdinands indexed and sub-indexed all work processes (practices that informants engaged in related to clothes shopping) and linked texts (e.g., size categorization systems, media advertisements) in the data contributing to this work. Through this inductive indexing process, Ferdinands developed the overarching categories of “learning work” and “performance work,” within which informants’ work was organized.

**Fig. 1:**
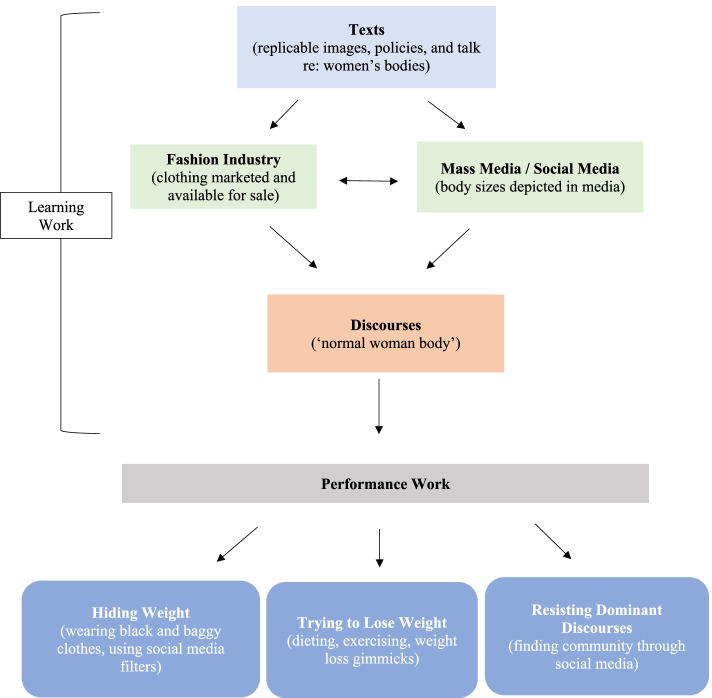
Young Women’s Weight Work Organized by Text-Mediated Discourses in Fashion and Media

Ferdinands also used mapping, in both the metaphorical written and diagrammatic sense, to illustrate the social organization of informants’ work [60]. As part of the analytic process, she regularly sketched out maps on paper depicting how informants’ work was linked to ruling relations. These maps, which served to guide our analytic writing, were continuously refined as more data were collected and more time was spent engaging with existing data. Figure 1 represents the “final” map.

### Study quality

We referred to Kiyimba et al.’s quality criteria throughout this study [61]. To promote *transparency,* Ferdinands maintained an audit trail by tracking research decisions and discoveries. *Transferability* occurs through uncovering generalizing social relations that organize local experiences. Prolonged engagement with five informants who participated in both individual and group interviews, and with two informant-researchers in the YouTube investigation, over several months increased the likelihood of generating rich data. *Ethicality* was critical, given we were exploring a sensitive topic with young women. Ferdinands obtained written consent at each in-person interaction (including parental consent for youth under 18) and observed informants’ body language for nonverbal cues suggesting discomfort. A study with *integrity* is methodologically cohesive, meaning all research components aligned with IE’s ontology and epistemology. Finally, *reflexivity* encompasses the self-appraisal of how our social positions positively and/or negatively shape research processes and results. Ferdinands considered how her identity traits (e.g., white, cisgender woman, thin, able-bodied, public health researcher) influenced the research focus and her relationship with the data, participants, and analysis. This included asking self-reflexive questions like, “who am I to share this account? What responsibilities do I have in doing so?” Despite striving to know the nuances of living in a larger body, she had to acknowledge she could not ever fully understand the embodied experience of being fat [62]. Rapport building may have initially been hindered by her lack of experience in a larger body, but the rich data generated suggest informants felt relatively relaxed sharing personal details, perhaps owing to her genuine curiosity in learning from them as experts of their lives.

## Findings

For context, research participants’ demographic information is provided in Table 1. The country or countries (for informants who moved during their childhood or adolescence) where informants grew up is included, due to the influence of geographical location on social norms around weight. In some geographical contexts, fatness may be preferable to thinness [63]. We did not formally collect additional demographic details, like race, sexual orientation, and (dis)ability, from informants, although we did discuss them if/when they surfaced spontaneously during interviews. These characteristics are not reported to protect informants’ anonymity and to align with IE’s core tenets. Smith contends that in taking abstract categories like race for granted, we jeopardize our ability to examine how people enter the social relations embedded in those categories [64]. Unless otherwise stated, all quotes below are derived from our individual interviews.

Figure 1 maps out this study’s findings. Youth weight work was broadly divided into two categories: learning work and performance work. We begin by describing how informants learned about weight work, and then explain how they acted on this knowledge, whether that was aligning with or resisting it.

### Learning about weight work

#### From media

##### Seeking, but not finding, body size diversity in media

From the moment informants were exposed to images of people on screens or print media, they noticed that only thin women’s bodies were displayed. It was difficult to find heavier characters to relate to in television or movies. Maria said:

Mostly every show I grew up with, the people are usually skinny. I can think of Raven from *That’s So Raven* that wasn’t stereotypically skinny. Or maybe from *Grey’s Anatomy,* the earlier seasons, Callie, she wasn’t stereotypically skinny. It’s just recent that you’re seeing more plus size characters like *This is Us*, especially as lead characters. And romantic interests, that’s not something you usually see. … It’s almost like they don’t allow for women who are plus size to, I guess not exist, but say that they do exist.

Jasmine felt there was a gender double standard: men could be heavier on-screen, but women could not: “There’s not that many heavier people [on television]. On top of that, I feel you’ll still see heavier men in shows, but you won’t really see a heavier girl, unless she’s supposed to be the butt of a joke.” Amy further explained these gendered norms: “I think women are more influenced by their body. Like their body influences their experiences more than men’s bodies influence their experiences.”

Similarly, our YouTube investigation reflected the notion that women faced stricter norms than men which led to greater emotional work on their behalf. Thirty-six of 45 videos reviewed exclusively depicted girls and/or women sharing conversation and concern around weight, whereas only seven of 45 videos reviewed exclusively portrayed boys and/or men. In this way, weight management was the work of girls and women; it was their responsibility to fit in socially. Through the YouTube videos and comments reviewed, it became apparent that users fostered community around how to cope with this “problem” through performance work discussed later in this manuscript.

##### Recognizing fat stereotypes

On the rare occasion that fat people were on screen, they were presented in a stereotypical manner. Sharmeen said, “you look at movies and the main characters are always gonna be thin, always gonna be pretty. They’ll have the weird character as fat, … eating McDonald’s every single day.” Emma furthered this: “When they do portray a bigger person, they’re always a specific weight, they’re always really loud. … If you’re fat, you kind of have only one choice. Like you have to be the funny friend if you’re fat.”

The “obesity epidemic” pictured on newscasts perpetuated these stereotypes. Angela expressed her concerns with the portrayal of fatness in health-related media:

If you ever see on the news like *Health Matters*, they talk about the obesity epidemic and they film random people on the street without showing their faces. … It absolutely terrifies me because I’m always worried I’ll be one of those people. I don’t like the way that it’s talked about as if this is pathological.

##### Feeling ashamed

Interacting with popular culture through media commonly left informants feeling inadequate. Christine said, “you just wanna look like the Instagram model or like the girl that you just look up to. Like she’s just better than you. At everything.” Maria described how observing her friends’ Facebook photos made her feel ashamed of her own body: “Growing up, [I couldn’t] help compare, especially to my female classmates. Who’ve been skinny as long as I knew them. … Especially since like in Mexico it’s hot, so you always see them posting pictures in swimsuits.”

Like Maria, Jasmine described the work of body size comparison, but in relation to celebrities rather than peers:

I was talking to my friend ‘cause this [K-pop] group just had a comeback this morning. So they put out a new album and first comment out of her mouth, she’s like “I’m not eating ever again.” ... There’s an unspoken rule in Korean society where if you’re over 50kg, you’re considered fat. I’m like, are you kidding me? [laughing] Am I a whale? … I read this thing recently like Miss Korea she was 68kg and people are like “can she be considered beautiful then?”

Weight norms differ across geographies, with thinness being an even stricter requirement for social conformity in Korea than Canada [65]. One YouTube video reviewed, “Women’s Ideal Body Types Around the World,” showcased these variations [66].

For many informants, interacting with social media was so stressful that they avoided it altogether, deleting social media apps from their phones, or selectively following accounts that would not make them feel badly about their bodies. For example, Angela said, “I just follow cats [on Instagram]”.

#### From fashion

Mirroring the lack of representation in media, informants explained how bigger people were socially excluded in the context of fashion talk and shopping among their peers. Fashionable clothes were not available for their body types. Emma explained how disheartening this was: “It’s not a great feeling, where all your friends are wearing the same things. My friends would talk about shopping at Lululemon or Gucci or something—like I can’t fit in any of that.” Bree echoed these frustrations: “When someone is like ‘oh you’re not fat, you’re perfect,’ you know what? I want to be able to go into a store and find clothes that I like, instead of clothes that actually fit me.” Angela described the emotional work of clothes shopping:

For bigger people there are not a lot of clothing options. And if there are, they just don’t look good. I would always hate clothes shopping, because I would need to try on five different things to find something that fit me. I have done a lot of crying in trying rooms because nothing would fit me.

Maria elaborated:

I don’t like shopping usually ‘cause they don’t have my sizes. Or if I go to a plus-size store like Torrid, it’s a really nice store. But the clothes don’t fit me ‘cause my boobs are smaller. Which is good they have that for plus size women with bigger boobs. But having pretty small boobs and being bigger, it’s hard to find clothes that look good.

In Maria’s experience, designers made assumptions about the dimensions of a “normal woman body”, negating the possibility for variation within. Likewise, Bree shared her experience of not being able to fit into what stores labelled “children’s clothes”:

One girl used to tell me, “why do you wear adult clothes?” I didn’t know what to say to her ‘cause I was just like “oh, it’s just my style”. But I knew that’s the type of clothes that fit me. The little pretty Mickey Mouse clothes you’re wearing, those just don’t fit me. ... I have to buy this ‘cause I don’t really have the choice.

Again, societal assumptions delineated what size a child should be. Lauren also felt, as a child, it was embarrassing to be the same size as an adult—her aunt:

My aunt always gives me, you know, those hand me down clothes and stuff. And she’s as well a bigger size. So that was very discouraging. I’m like okay, so now I am matching her size. … When it gets to a point where oh no, my size is bigger than let’s say my friend. Or my mom. Or my sister. Then I start to question, okay, I guess I have a problem. I’m not blending in the norm.

Group interviews revealed informants’ insight into the challenges of selling plus-sized clothing:

Sarah: Nike had their first plus size mannequin and it got a lot of backlash. Calvin Klein had their first plus size model, got a lot of backlash. Victoria’s Secret also had their plus size model, got a lot of backlash. So there is backlash from the public itself. Even if companies are trying to change as well.

Angela: In those cases, I mean I don’t know about all of those kinds, but in at least one of them I think, the plus size model was literally just a normal woman.

Christine: It’s a double-edged sword ‘cause it’s like, oh she’s not big enough, but they’re also like, if you make her bigger then you’re just promoting obesity. So there’s no winning. … If you don’t put a plus size model then you get shamed for being sizeist because you’re not inclusive to bigger people.

The group also discussed discrimination in terms of plus-size clothing costs:

Bree: One day at Walmart I noticed a sign being like, I don’t remember how many dollars more being a bigger size was, I was just like why do people have to pay more for a bigger size? That was the first time I saw that. I was like, I don’t get it. If you don’t have to pay more from small to medium, why does someone have to pay more from an XL to XXL?

Alexa: Was that here in Canada?

Sarah: Yeah it was in Edmonton actually.

Christine: Really? I felt that there’s a law on that. ‘Cause that’s discriminatory.

Bree: I know!

Christine: Even though you’re spending more money on fabrics because you’re paying more for a bigger size, you shouldn’t discriminate. …

Sarah: I think that happens at H&M too … when there’s one size left, it’s [cost] usually higher if the size left is bigger than if the size left is smaller.

Lauren reflected on the dehumanizing aspect of clothing size categories:

I find it really annoying with those clothing sizes, you know like zero … double zero, XXL, wow. … You are the S size. You are the medium size. You know. We are categorized in these sizes—that’s all we are.

Sarah noted her frustrations with size variations between brands, particularly when her clothing size was not available at all:

I don’t like how you always have to check the sizing chart. … I’m gonna shop at Ardene and I check the sizing chart and I’m like, I don’t fit anywhere. Not even an XL. In another clothing store I would but like here, I’m just like, okay goodbye now.

With her postsecondary background in textiles, Christine offered unique insight into the fashion industry’s approach to sizing: “Some brands have different size zeros or smalls are mediums. You know, there’s no standardized thing. A lot of the time, it’s there to make you feel worse about yourself.” Because participants grew up in countries around the world (Table 1), they knew sizing systems differed internationally. Group interview informants conceded that in Canada, clothing sizes were more generous than in their home countries. Bree elaborated:

Back in Mauritius, the sizes are smaller ‘cause I think a lot is from China and stuff. So I was a large. Just saying back home, like oh a large, an extra-large made me feel bad because I’d feel the person would judge me. … But here, when I say I’m a medium, I feel normal.

The notion of “feeling normal” correlated with having a body size conforming to social standards. Christine enlightened fellow group interviewees of fashion design protocol which perpetuated unrealistic body size standards:

When you’re sketching models, you don’t sketch bigger people. The standard is eight heads, so one head and you have to do it eight times. Or no, actually it’s nine heads. You make it absolutely proportional to everything. So your waist is three heads down. And then your legs are five heads. … These women I’m drawing are disgustingly skinny. … Designers and stuff like that [thinness], so of course they’re not having good designers making plus-size clothing.

Overall, through text-mediated discourses permeating fashion and media, informants learned the importance of and strategies for performing weight work—whether that be aligning with or rejecting dominant discourses around what it means to have a normal woman body.

### Performing weight work: aligning with dominant discourses

#### Trying to lose weight

All informants expressed and acted upon a desire to lose weight to achieve the normal woman body. Many commercial industries have capitalized on this desire, including the diet, exercise, pharmaceutical, and “miscellaneous gimmicks” industries. We examine how informants interacted with these industries below.

##### Dieting

Eating practices are regularly cited as a “lifestyle risk factor” in public health discourse. It follows that dieting was the most common approach to weight loss among informants, understood as something that all girls and women should do, regardless of their body size. Angela began dieting in elementary school:

Starting from when I was eight, I would try and go a whole day without eating. It was very, very unhealthy. Because my only input of weight was from people that I would talk to and then TV would have like “oh try this new diet”. When I was nine it was my dream to go on Jenny Craig. Very sad.

Sharmeen felt most diets were unsustainable. She did not label or offer detail about the diets she tried, but referred to dieting generally, implying eating healthfully was common sense:

I’ve definitely tried to eat healthier. Like I will eat salad and I still do. If there’s chicken right, I’ll just eat that. But then also I’ll be like ‘kay, I’m only gonna eat a salad after school. But then I get home and I’m like, I’m hungry. You know? And not for a salad! … So I’ll start it but I’ll never follow through with it.

Informants pointed out that the evidence behind many diets was flawed. Christine joked about the weight loss myths circulating in her household.

She’s [mother] believed that you know putting all these fruit scraps together … if you make a tea out of it, you’ll lose weight. It’s disgusting. It’ll be banana peels, onion stuff, strawberry scraps, all this stuff. … She goes on the internet all the time to find these things and watches a lot of TV, that’s like “oh yeah, this is so healthy”. She has a little book and writes all this stuff down.

While Christine mocked her mother’s reliance on unfounded weight loss strategies, she too went on crash (i.e., severely calorie restricted) diets. Apparently, the social push to be thin superseded logic. Ready access to the internet and mobile apps (such as MyFitnessPal™, a calorie tracking app) enabled the diet industry to pervade young people’s lives.

The marketing and sales of diet products were prominent in conversations with informants. Eden recounted the body weight surveillance she endured as a child by her friend’s mother at swimming practice. This mother, feigning concern for Eden’s health, repeatedly offered her unsolicited advice, encouraging her to buy products from Isagenix, for which she was a sales representative. Isagenix is a nutrition company selling diet products, ranging from pills, to shakes, to supplements.

She’s [mother] very pushy about it. Even as I was swimming, she would always go to my mom and be like “oh she shouldn’t be drinking Gatorade, she should try this Isagenix thing.” … Every time she talked to me, she was like “you should be doing this, oh this will help you lose weight”.

Altogether, there were two forms of dieting marketed to informants: 1) food, macronutrient, and/or calorie restriction; 2) dieting gimmicks, like metabolism-boosting, fat-burning supplements.

##### Exercising

Exercise goes hand in hand with dieting in public health discourse as a conventional weight loss approach. When we asked Jasmine why she kept going to the gym when she disliked it, she responded: “well my mom keeps telling me to go”. Lauren’s parents too encouraged her to exercise to lose weight, which she described as: “typical traditional thinking, you know. Do more exercise.”

Although exercise was cited in dominant discourses as a means of achieving weight loss, informants felt unwelcome in fitness venues, fearing surveillance. Bree said she was not “comfortable enough to go where the Instagram fitness models go”. Many informants felt exercise should be confined to their bedrooms or basements where they would not be seen, relying on at-home spin bikes and YouTube exercise videos to achieve thinness. Maria elaborated: “I hated gym class. I don’t like going to the gym; it makes me really self-conscious. I get really sweaty and really hot and red when I exercise. I don’t like doing that in front of people.” Informants felt especially uncomfortable exercising in the vicinity of men, who commented on their supposedly incorrect use of equipment, improper form, and so on. Bree explained:

A lot of people don’t want to go to the gym because they feel scared people are gonna judge them there. Especially the weights by the guys and stuff. It’s already hard for girls to go there ‘cause there’s so many guys. … You feel like this is not my place to be.

For these reasons, Lauren exclusively attended a women’s only gym.

##### Combining diet and exercise

Many weight loss programs informants referred to drew upon some combination of diet and exercise. For example, Christine described participating in a fitness challenge accompanied by a “crazy strict diet” in our group interviews. This six-week weight loss challenge was advertised on Facebook, promoted by XTherapy. Lauren also told us about the weight loss regimens she had tried while simultaneously managing Type 1 diabetes:

I sought out YouTube and started watching exercising videos and seeing what their diet plans are … These low carb diets, Atkins diet, paleo. … With the diabetes, I thought this way I could also reduce my insulin intake. … Of course, if you have too low carb, breaks down your muscles, so you’re just getting fat again. … I just felt I’m so unhealthy, I got to make some changes. But I don’t think you know following those low carb diets or any of those trendy diets are the way, definitely not.

Evidently Lauren faced a disjuncture: despite stating that these strategies were not “the way” to lose weight, she still felt obliged to engage in them.

Jessica’s account of attending a weight loss summer camp epitomized just how grueling these regimens can be. She flew to the United States from Canada for a weight loss summer camp lasting several weeks when she was 14. This camp cost her family several thousand dollars, when they had scarce financial resources to begin with. She reported experiencing “endless exhaustion” at camp, but enjoyed it because she lost 50 pounds that summer, and hoped to return the follow summer to “get back on track”.

Motivational messages to lose weight through diet and exercise were common on YouTube. Thirteen of 45 videos reviewed explicitly or implicitly encouraged viewers to lose weight. Extreme weight loss was depicted as inspirational, with an emphasis on achieving it “naturally”. One of the most concerning examples was entitled “12 year old weight loss transformation: my weight loss journey” from the Daily Life of Lexie YouTube channel [67]. Lexie, the 12-year-old narrator, shows the audience how she hated her body (including images of her prodding her belly fat) and so began an intense diet and exercise regimen to lose weight. Video clips include Lexie wagging her finger “no” at a Pizza Hut box, while giving a thumbs up to strawberries and broccoli. After enduring rigorous workouts on gym equipment unsuitable for a 12-year-old, she steps on the scale at the video’s end, appearing elated to no longer have a “double chin”.

##### Taking the “easy way out”

Diet and exercise were deemed valiant weight loss tactics, representing willpower and hard work. But informants also shyly reported using a range of alternative weight loss products. The shame surrounding these products relates to how they reinforce stereotypes that heavier people are lazy. Perceived as taking the easy way out, such products are used by those who lack the moral fortitude to lose weight “properly” (i.e., “naturally”) through diet and exercise. At a young age with no personal income, informants relied on their parents to buy such products. Several years ago, Bree asked her mother to buy weight loss products, like body wraps to shed belly fat. Her mother bought them, but now, Bree felt embarrassed about having wasted money and time on these products.

While no informants used weight loss pharmaceuticals like Saxenda®, the topic arose during our group interviews:

Christine: My boyfriend’s mom, her doctor’s convinced her to do a thing where instead of actually working out or changing your diet, she just takes injections. … She was complaining she paid eight hundred dollars for these thirty injections that make her lose weight, and then she’s not losing as much weight as anticipated. …

Angela: I am gonna make a guess and tell me if I’m right. Those injections, were they marketed specifically by that clinic?

Christine: I don’t know … all around their clinic was like [ads], “are you overweight? Try talking to your doctor about these injections, see if they work for you”. So, maybe. I thought it was weird and you know, of course insurance does not cover that kinda stuff because it’s something you want, not something you need.

Bree: They make it this insecurity and just make money out of it. That’s pretty messed up ‘cause you trust your doctor to give you a solution that works.

This conversation touched on many facets of the ruling relations. Informants felt doctors promoted certain drugs as part of some money-making scheme. We were surprised by Christine’s remark of weight loss pharmaceuticals as “something you want, not something you need” given that she, and others in the group, had experienced firsthand the difficulty of weight loss. But they still interpreted taking medication as cheating. Bariatric surgery is also commonly seen as a dishonourable route to weight loss [68]. No informants had had bariatric surgery, but Emma was considering it after her family doctor had suggested it.

#### Trying to hide weight

Hiding the shape and size of informants’ bodies was primarily achieved through their clothing choices. Lauren described her tendency towards “loose clothing. Nothing tight that will just reveal fat. Or wear all black all the time because that’s what makes you look skinnier.” Larger people are taught to stick to black clothing, avoiding patterns or striking colors because those draw attention to their size. The ruling relations dictated that informants could not choose clothes freely. YouTube videos, such as “Curvy Outfits Dos & Don’ts! 10 Style Hacks for a Curvy Body!” [69], instructed bigger viewers how to dress in a socially acceptable manner.

Sharmeen described how she deflected attention from her body:

I started to wear not *really* baggy but clothes that weren’t formfitting. … My best friend, she’s very thin. And she always wears really nice and “out there” clothes. Formfitting and different styles. I wanna do that. But if I think of an outfit in my head … I’ll try it on and be like oh, no, never. So I just stick with my hoodies and things. It’s kinda sad ‘cause I feel like I can’t explore more.

Maria explained how her clothing preferences changed with her weight:

I stay towards a certain type of clothes … a baggy type I feel more comfortable in. But it does hide my body more. I used to like tighter clothes, like crop tops. I liked those until recently since I gained more weight.

Garments required for sports influenced informants’ ability to participate. Christine explained how she felt like she was not allowed to swim:

I really hate wearing bathing suits because I’m exposing so much of my body. … I feel if you wear a shirt over yourself, you’re not confident with how you look and you don’t want other people to see. Which is exactly what I’m feeling. So I really don’t like going into the water.

Jessica dropped out of dance classes as a child due to a similar discomfort:

It’s a lot of body image issues with dance because of the costumes that are accentuating that sort of perfect body type … skin-tight body suits and everything.

Christine again shared her fashion design knowledge, this time around creating thin silhouettes.

You can always make the illusion of looking skinny but in the end it’s always like, you still wanna be as skinny as possible. … In high school that’s pretty much what I learned. How to either make yourself look skinnier, or if you’re too skinny, make yourself look a little bigger.

Here, Christine pointed out the difference between illusion and reality, noting that while feigning thinness was a good step, it was preferable to “actually” be thin.

Informants also attempted to hide their weight virtually. But when caught for doing so, it was embarrassing. For example, Sarah used face-slimming filters on social media apps like Snapchat and Instagram:

If I saw my face without a filter, I’d just make a joke about it. But there’d be times where I think people would see my Instagram profile and be like you know this is fake ‘cause of the filter. ‘Cause of how I look in real life.

Unfortunately, this event happened to Sarah. She overheard international students (young men) at university discussing (in a language they thought she didn’t know—but she did) how her Instagram profile looked fake. Humiliated, she took the picture down.

### Performing weight work: resisting dominant discourses

While trying to lose weight and hide fatness were the most common modes of performance work, the final mode involved resisting this dogma altogether. Not only did informants disrupt media representations of the normal woman body, they also challenged contradictory media representations of body positivity. For example, Emma said:

Even though they’re trying now to make it more apparent that there are different weights and stuff, when it comes to portraying people on TV or in ads, first of all making them different sizes. And actually different sizes. Not just like they do like “everybody is beautiful,” but then they’re all the same size.

While 13 of 45 YouTube videos had a title or purported intent suggesting content around body love and acceptance, their overwhelming message was still to lose weight. Moreover, some videos depicting body love were of categorically thin women “learning to love their curves”, potentially excluding women at higher body weights from these conversations. Notably, pressure to love or accept your body imposes another form of work on women—it becomes their responsibility to be content with their bodies despite societal messaging suggesting otherwise [70].

However, there were positive aspects of social media, including its ability to create space for community. Thirteen of 45 YouTube videos involved people sharing their experiences of being overweight, helping informants to feel less alone (e.g., “Being the fat girl at the gym” [71]). Using social media also opened informants’ eyes to new ways of thinking about body weight. For example, Bree described how she learned about reclaiming the term “fat” as a neutral descriptor from a YouTuber named Sierra: “She really helped me to understand that being fat is not a bad word. It’s just like you’re short or you’re tall. Maybe you’re thin, maybe you’re fat, it’s fine. That doesn’t have to be an insult.” Maria shared how the media shaped her thoughts about bodies:

It was internet where I started seeing more body positive things and people who are large and they love themselves. … Recently I was thinking I have to support more women of plus size since they were so underrepresented. So, if I saw a woman of plus size that showed up in my [Instagram] explore page and I saw what they were doing and I liked it, then I’m like, I need to support her so more women that are plus size are represented and they have more opportunities.

In fashion, Christine felt there have been some positive shifts in representing larger bodies, albeit modest: “Nowadays people are starting to embrace the plus size model which is great. I really appreciate that. Like it’s starting to move standards from anorexic skinny white model to more diversity.”

Overall, this resistance work demonstrated that young people were not puppets of the ruling relations—they were working to change them. But should young people in bigger bodies alone be burdened with this titanic task? As public health researchers invested in youth health promotion, this seemed an issue worth exploring.

## Discussion

Though published three decades ago, Smith’s description of women’s bodies as a laborious, life-long project continues to ring true [1]. Our findings support a vast body of critical weight and fat studies literature— fields with a rich history of critiquing neoliberal agendas which have constructed the fat body as problematic [5, 19, 72, 73]. This study found that texts replicated through the institutions of fashion and media, predominantly in the form of images, mediated discourses around the normal woman body (Fig. 1). These texts, activated by informants’ engagement with them, were so commonplace that informants learned to take for granted what women’s bodies of all ages “should” look like. The lack of larger women’s bodies witnessed in media and fashion discursively silenced informants, whose bodies were categorized as taking up too much space. Whether it be scrolling through fitness challenges on Facebook, following Photoshopped lifestyle influencers on Instagram, or noticing their mothers try various diets, informants learned they had to acquire a specific body type to be seen and heard. Informants responded to this actuality by performing three general types of work: hiding their weight, trying to lose weight, and resisting dominant discourses. Much of this work required engaging with commercial industries beyond those directly affiliated with media and fashion, including the businesses of diet and exercise.

The technology and diet industries have joined forces to create weight loss apps targeting children and youth such as Kurbo, developed by WW (formerly Weight Watchers). Various organizations have slammed Kurbo for its potential role in triggering eating disorders and reinforcing problematic, binary assumptions around good and bad foods [74, 75]. Food-like diet products, like Kardashian-endorsed Fit Tea, advertised through social media were also prominent in informants’ accounts. Young people spend large portions of their day plugged into the internet through mobile phones and computers; policies are needed to block social media advertisements for weight loss and dieting products from young users, as such those recently implemented by Instagram and Twitter [76, 77]. Banning Photoshopping of bodies in media, including fashion advertisements, is another strategy that could help to normalize body size and shape diversity.

One example of size inclusivity in fashion is a revolutionary initiative introduced by Old Navy across North America in August 2021—BODEQUALITY—which involved eradicating their women’s plus-size department; instead, integrating *all* sizes into the mainstream at the same price [78]. Old Navy promises that every women’s clothing item is available in sizes 0–28 online and in-store (with size 30 available online) [78]. How can public health professionals meaningfully engage with folks in the fashion industry to create or modify policies mandating equal price points for and consistent availability of larger clothing sizes, which would make examples like BODEQUALITY the norm, rather than an anomaly? And is meaningful engagement even possible in the face of consumer capitalism [79, 80]?

Notably, our discussions with informants about their experiences navigating social media suggested that content alleging body positivity often still focused on weight loss. Similarly, Bombak et al. describe how industries paid lip service to body positivity—a term which has been coopted as a “corporate buzzword” ([81] p. 199). Although informants pointed out some positive discursive change regarding body acceptance in media and fashion, this progress had little impact on how others treated them (and consequently, the work they are required to do) in their everyday lives. Findings suggest an interim need for coping tools to help young people thrive while “coming of age in toxic image environments” ([5] p. 244). Sturgess and Stinson propose “embracing the online fatosphere” as one approach to coping [82]. Our informants similarly alluded to how the fat-positive online community can provide a safe, empowering space for resisting societal weight stigma and internalizing a positive fat identity [82]. While such coping approaches may be band-aids rather than sustainable solutions, structural changes take time, and we cannot neglect those negatively affected in the here and now.

We would be remiss to overlook the complexity and uncertainty surrounding structural (i.e., policy and institutional) change [83]. In their description of an “inside-out” social ecological model, Golden et al. underscore that simplistic notions of structural change often fail to address how such changes actually happen [84]. Incorporating systems thinking concepts like feedback loops, they call for public health professionals to reflect on how collection action from individuals contributes to policy and institutional changes [84]. Their model overlaps with IE’s theoretical framework, in that it returns attention to people (including public health professionals) and the reverberating effects of our individual actions (our “work”) [84]. Such actions include thinking critically about the language and images we use in knowledge translation (acknowledging the impact of enacting texts in shaping our realities), or challenging microaggressions to indicate what we view as socially acceptable behavior. Meadows et al. emphasize that “while we continue to advocate for policy change at the macro level, we must also continue to challenge fatphobia when it occurs in our daily lives and interactions” ([39] p. 118).

### Strengths and limitations

To our knowledge, this is the first study employing IE to examine public health orthodoxy around obesity. While many public health researchers have examined weight stigma as a theoretical construct, comparatively little work has been done to examine its materialistic constitution; that is, how it is coordinated to happen. Starting our inquiry by examining young women’s work, and consistently prioritizing their standpoint throughout the investigation, helped to ground our analysis in the actual everyday world, keep our informants in view, and notice particularities of their work that may otherwise be ignored. Strengths of this study include the original maneuver of eliciting discussion with informant-researchers by collaboratively reflecting on their investigation of YouTube videos, wherein we viewed how the replication, or lack thereof, of particular images played a key role in socially organizing informants’ weight experiences. We also generated a large amount of data in this IE project, which rendered the analysis complex, but increased the likelihood of having rich data to draw from. Another strength is that we privileged the standpoint of young women. Much research has ignored their voices. Hiring informant-researchers to guide and participate in our YouTube investigation was a unique and empowering approach for the young women.

In this study, we exclusively spoke with standpoint informants; future research would benefit from conversations with professionals employed in fashion and media to examine their work and how it intersects with that of the standpoint informants. Although we spoke with young women with diverse lived experiences, there was limited representation from many groups, like people with disabilities. Therefore, other examples of resistance to dominant discourses may be missed. Further, we only interviewed cisgender youth and did not explicitly probe about their sexuality. Research is needed to explore the weight work of sexual and gender minority youth, who may be at higher risk of weight-based victimization [85]. While we did interview women who had grown up in countries around the world (Table 1), a greater focus on racial and ethnic social relations from the beginning stages of our inquiry would have facilitated deeper exploration of how weight discourses are applied across different racial and ethnic groups. Most informants were university students; their experiences may not be the same as those who chose not to or were unable to attain postsecondary education for reasons including socioeconomic deprivation. Our recruitment posters used medicalized language (“overweight”, “obese”) which may have restricted the types of individuals who opted to participate, given that they would have had to identify with a body labelled this way. Future research should use more inclusive language (e.g., “larger,” “heavier”). We also acknowledge that by not disclosing informants’ relative body sizes, we are limited in sharing important aspects of informants’ experiences with fashion, imagery, and the types of body work required of them. Future research should ask informants to self-identify such that this information can be analyzed and reported on. Because this project was conducted as an exploratory overview of informants’ interactions with fashion and media, a deeper inquiry following the threads of each of these institutions are warranted.

## Conclusions

In this study, we showed how the consistent lack of larger bodies in the fashion and media images that young women were exposed to throughout their growing up significantly shaped their understanding of what it meant to have a normal body. This embodied knowledge urged young women to remediate their purportedly defective bodies. For no informants did this work ever end. While academic public health discourses may be slowly evolving to acknowledge both environmental influences on weight and the harms of weight stigma, general public discourses (including those tied to fashion and media) have yet to catch up. Public health professionals must continue to pay attention to how the institutions of fashion and media complement that of public health in shaping weight discourses, to broaden our thinking around how weight discourses are (re)produced and uncover how current ways of talking and thinking about weight and stigma may limit our interpretations of causes and solutions. Intersectoral collaboration is needed to move past preaching to the (public health) choir and stimulate innovative approaches for mobilizing weight stigma evidence beyond academic circles into our everyday practices.

## Supplementary Information

**Additional file 1. **Youtube data collection guide.

## Data Availability

The datasets generated and analyzed during this study are not publicly available to preserve informants’ confidentiality but are available from the corresponding author upon reasonable request.

## Abbreviation

IE: Institutional ethnography
