# Supplementary material for: “We’re categorized in these sizes—that’s all we are”: uncovering the social organization of young women’s weight work through media and fashion
Source: BMC Public Health. 2022 Jun 15;22:1193. doi: 10.1186/s12889-022-13607-w (PMC9199247; doi:10.1186/s12889-022-13607-w)
Supplement: Supplementary file 1 — Additional file 1. Youtube data collection guide. [file 12889_2022_13607_MOESM1_ESM.docx]

**YOUTUBE DATA COLLECTION GUIDE**

1. Date of viewing:
2. Video title:
3. URL:
4. How many times has this video been viewed?
5. When was the video released on YouTube?
6. How many thumbs up (likes) does this video have?
7. How many thumbs down (dislikes) does this video have?
8. Observe the people in the video.
   1. What is their ethnicity/race?
   2. Gender?
   3. Estimated age?
9. In one or two sentences, what is this video about?
10. On a scale from 1 to 10, how much did you like this video? (1=hated it, 10=loved it)
11. Did you like anything in this video? If so, what?
12. Did you dislike anything in this video? If so, what?
13. What kinds of words do people in this video use to describe body size? (e.g., fat, obese, heavy, big)
14. How does this video make you feel about your body, and other bodies?
15. What does this video say about body size, if anything?
16. Would you recommend this video to your friends? Why or why not?
17. Are any fat stigmatizing comments written about this video (limit observations to the first twenty comments made)? If so, what do they say?
